# Supplementary material for: APC2 controls dendrite development by promoting microtubule dynamics
Source: Nat Commun. 2018 Jul 17;9:2773. doi: 10.1038/s41467-018-05124-5 (PMC6050278; doi:10.1038/s41467-018-05124-5)
Supplement: Supplementary file 1 — Supplementary Information [file 41467_2018_5124_MOESM1_ESM.pdf]

Supplementary information

APC2 controls dendrite development by promoting microtubule dynamics

by Kahn, *et al.*

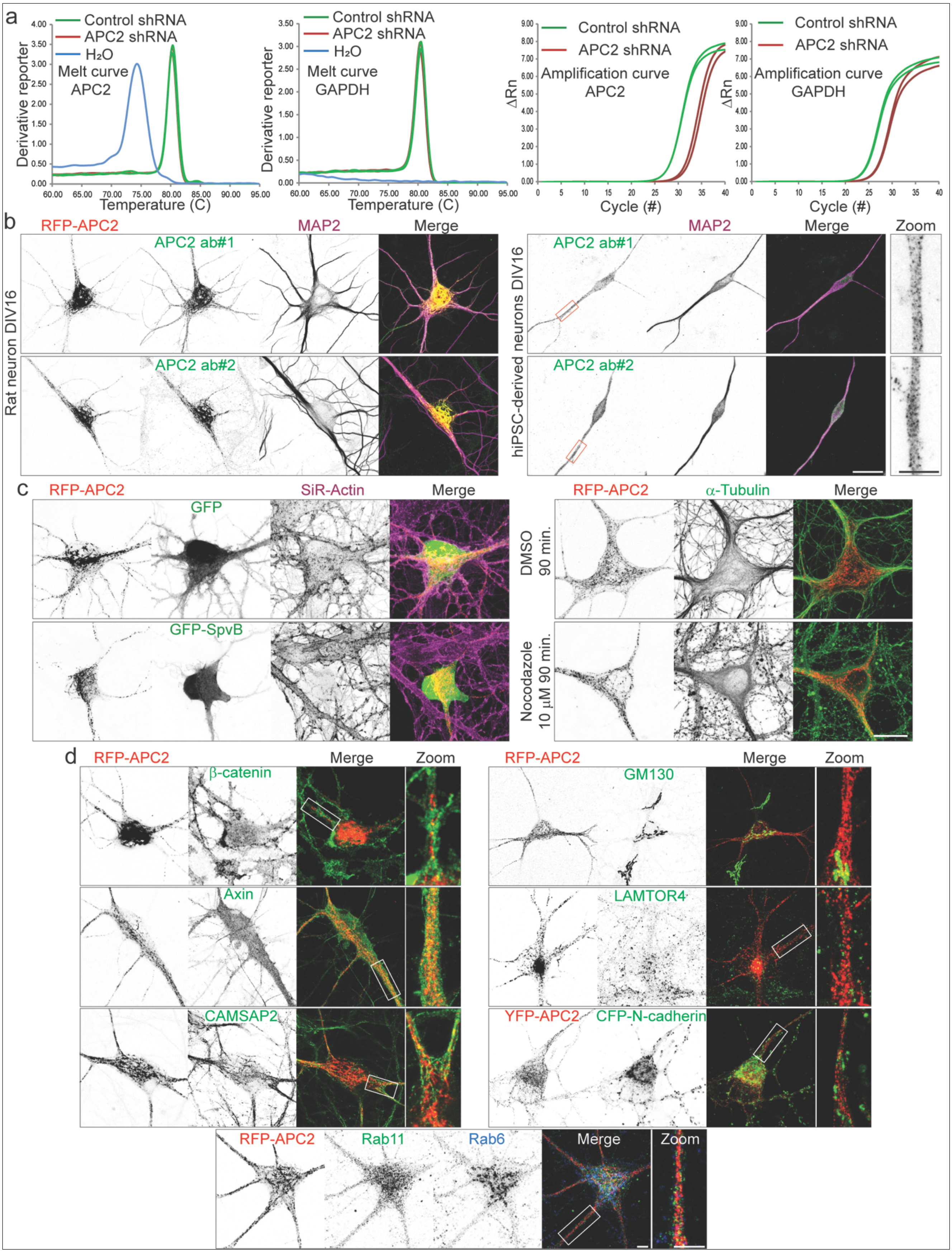

Supplementary Figure 1. APC2 characterization. **a.** Representative qPCR profiles for GAPDH and APC2 mRNA in control or APC2 depleted rat hippocampal neurons. Melt curves for APC2 and GAPDH products and amplification curves for one of the qPCR experiments are shown. **b.** RFP-APC2 was expressed in rat hippocampal neurons, fixed 24 h later at DIV 16 and immunostained for one of two different antibodies against human APC2 and for MAP2. Human iPSC-derived neurons were fixed at DIV 16 and immunostained for one of two different antibodies against human APC2 and for MAP2. **c.**

Representative images of effect of GFP-SpvB on RFP-APC2 and actin and Nocodazole treatment on RFP-APC2 and tubulin in DIV 15 hippocampal neurons. **d.** Neurons were fixed after 24 h of expression of RFP-APC2 and immunostained for β-catenin, Axin, CAMSAP2, Rab6, Rab11 and LAMTOR4. CFP-N-cadherin was co-expressed with RFP-ACP2 and neurons were fixed after 24 h of expression. Scale bar is 10 μm in **b** and 5 μm in **b** zoom, **c** and **d**.

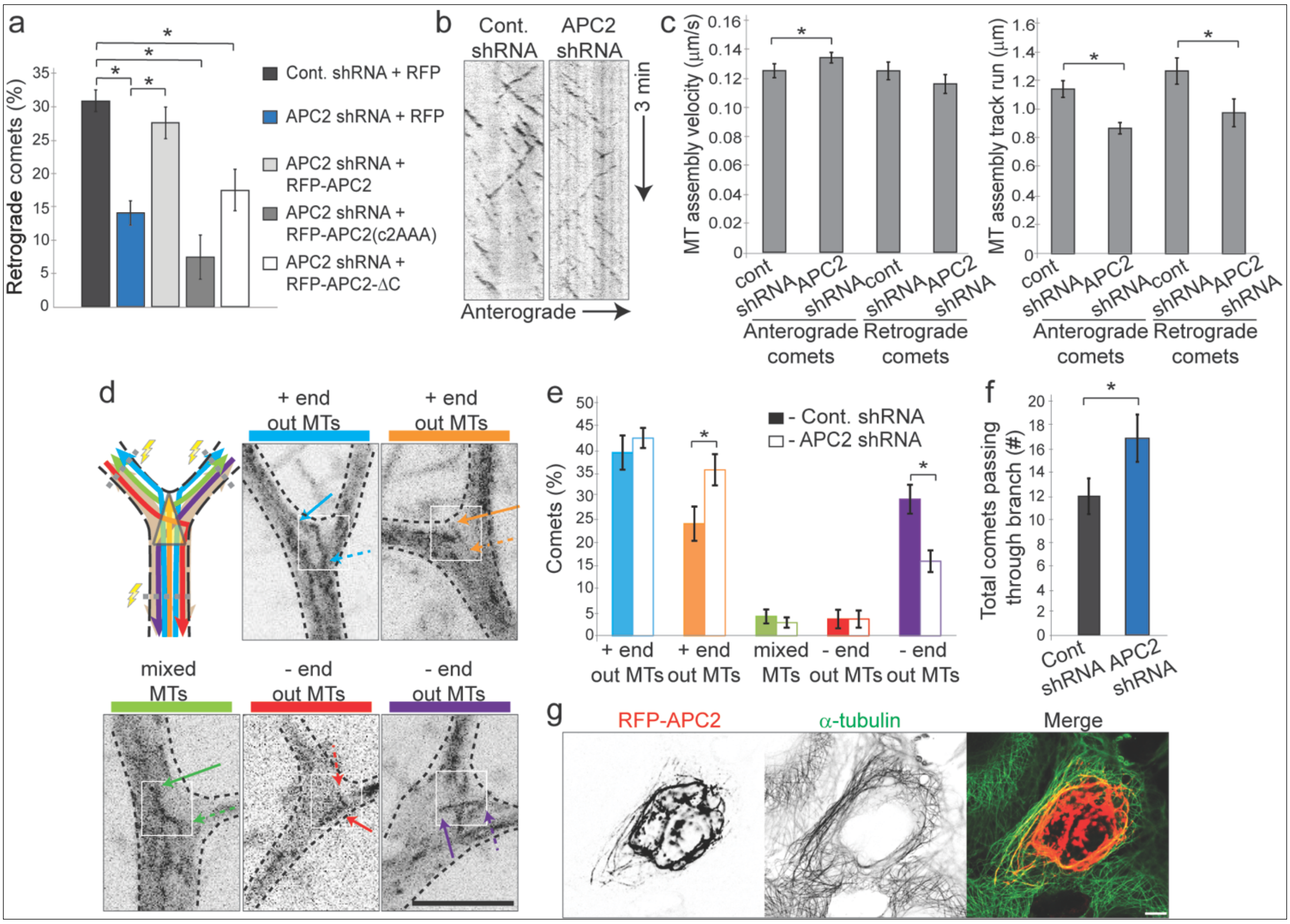

Supplementary Figure 2. APC2 effect on microtubule polymerization and steering at branch points. **a.** Percentage +TIP comets assembling in dendrites toward cell body in neurons transfected with either control or APC2 shRNA together with either RFP, RFP-APC2, RFP-APC2-ΔC or RFP-APC2(c2AAA) and imaged at DIV 15-16 (n=42 dendrites control shRNA+RFP, n=41 dendrites APC2 shRNA+RFP, n=15 dendrites APC2 shRNA+RFP-APC2, n=14 dendrites APC2 shRNA+RFP-APC2-ΔC, n=10 dendrites APC2 shRNA+RFP-APC2(c2AAA), N=4, One-Way Anova, Tukey post-hoc). **b.** Neurons were transfected with GFP-MT+TIP and either control or APC2 shRNA on DIV 11-12 and imaged on DIV 15-16. Representative kymographs of 3 min movies with frames taken every 0.5 sec are shown. **c.** Velocity of microtubule assembly (n=17 dendrites control shRNA, n=16 dendrites APC2 shRNA, Mann–Whitney U test) and track length was measured to calculate length of one polymerization event (n=17 dendrites control shRNA, n=16 dendrites APC2 shRNA, N=4, Mann–Whitney U test). **d.** Neurons were transfected with GFP-MT+TIP and either control or APC2 shRNA on DIV 17 and imaged on DIV 21. First primary dendrite branch point was used to quantify growing microtubule steering. Microtubules were photo-ablated prior to and post branch point as shown in the diagram. Microtubule growth patterns were classified into 5 color-coded paths within the yellow triangle from 5 min movies taken every 0.5 sec directly after photo-ablation. Maximum projections are presented showing specific tracks. Dashed arrow specifies beginning of track, solid arrow specifies the end of track. The five tracks were classified as follows: blue – plus-end-out microtubule polymerization sharply steered into one or the other branch, orange – plus-end-out microtubule polymerizing into the branch point and stopping, green – minus-end-out microtubule polymerization steered from one branch into the other branch, red – minus-end-out microtubule polymerizing into opposite periphery of dendritic shaft and sharply steered towards cell body, and purple – minus-end-out microtubule gradually polymerizing across the branch point towards cell body. **e.** Percentage of total comets passing through the yellow triangle in 5 min was quantified and classified into 5 microtubule growing paths. Solid color bars control shRNA and empty color bars are APC2 shRNA (n=18 branches control shRNA, n=15 branches APC2 shRNA, N=3, Independent samples T-test). **f.** Total comet number passing though the yellow triangle was also quantified (n=18 branches control shRNA, n=15 branches APC2 shRNA, N=3, Mann–Whitney U test). **g.** Representative image of overexpressed RFP-APC2 in Cos7 cell. Graphs represent mean ± SEM. * $p<0.05$. Scale bars are 5μm.

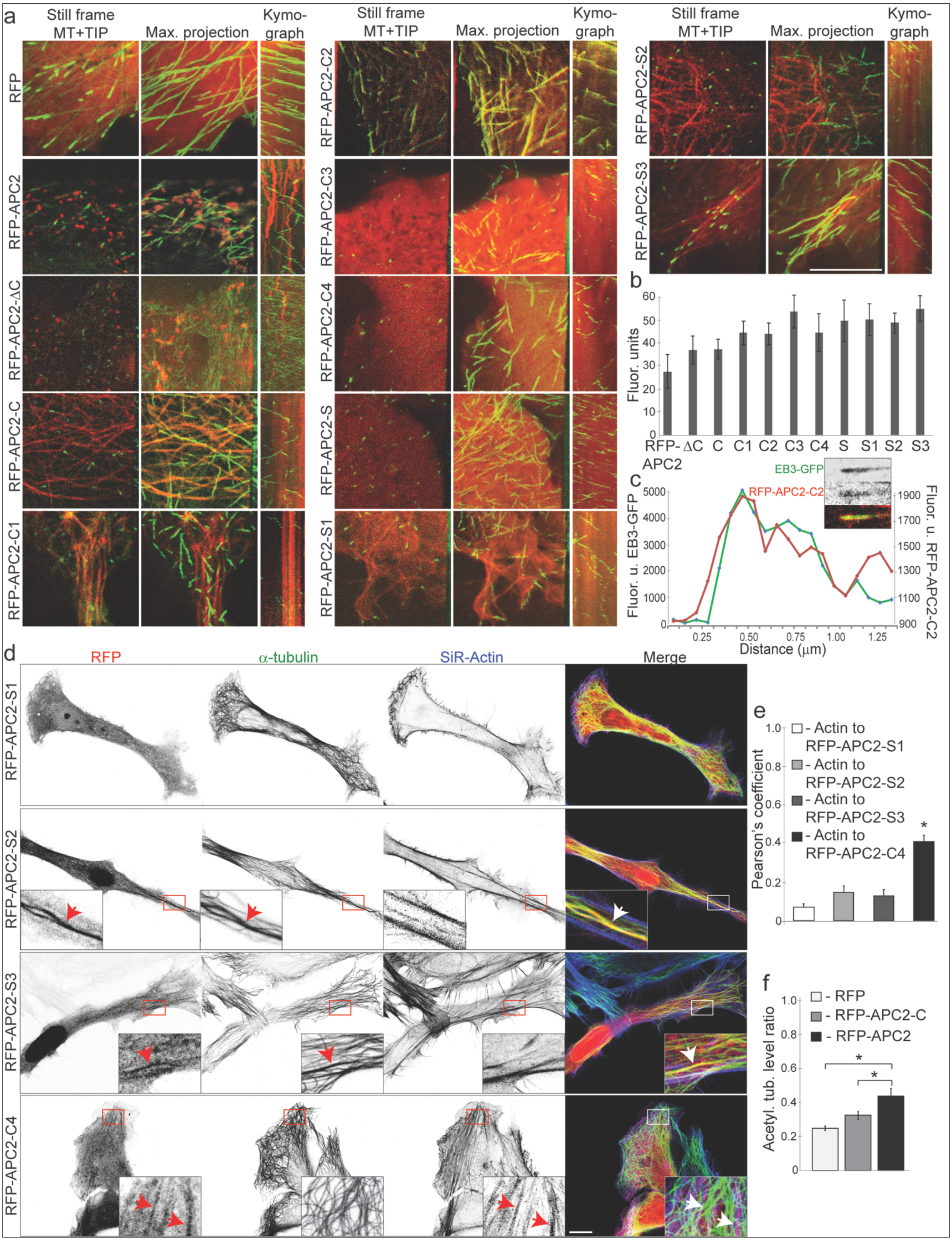

Supplementary Figure 3. Functional domains of APC2 cytoskeleton interacting region. **a.** Cos7 cells were live-imaged after 24 h expression of GFP-MT+TIP and the specified constructs. In the case of RFP control and RFP-APC2-C2 the +TIP co-expressed was EB3-GFP, the rest of the truncations were co-expressed with MACF18-GFP. Maximum projections are of 50 frames taken every 0.5 sec. **b.** Fluorescence intensity of RFP-APC2 truncations was measured after 24 h of expression in Cos7 cells to ensure equal levels of expression. Max 2 areas per cell were measured with 10x10 μm ROIs (n=21 areas

for RFP-APC2, RFP-APC2-ΔC, RFP-APC2-C2, RFP-APC2-C3, RFP-APC2-C4, RFP-APC2-S, RFP-APC2-S2, n=46 areas for RFP-APC2-C, n=55 areas for RFP-APC2-C1, n=36 areas for RFP-APC2-S1 and RFP-APC2-S3, N=2, One-Way-ANOVA, Tukey post-hoc). **c.** Line scans of a single microtubule polymerization event co-expressing EB3-GFP and RFP-APC2-C2. **d.** HeLa cells were fixed after 24 h expression of the specified constructs and immunostained for tubulin and actin. Arrows point to colocalized pixels. **e.** Pearson's coefficient was quantified between actin and either RFP-APC2-S1, RFP-APC2-S2, RFP-APC2-S3 or RFP-APC2-C4 from max 2 areas per cell, 10x10 μm ROIs (n=16, N=2, One-Way ANOVA, Tukey post-hoc). **f.** Cos7 cells were transfected with RFP, RFP-APC2, or RFP-APC2-C and immunostained for acetylated and α-tubulin. Whole cell fluorescence levels of acetylated tubulin where ratio was take over total tubulin was quantified (n=27 cells RFP, n=34 cells RFP-APC2, n=39 cells RFP-APC2-C, N=2, One-Way ANOVA, Tukey post-hoc). Graphs represent mean ± SEM. * $p<0.05$. Scale bars are 10 μm.

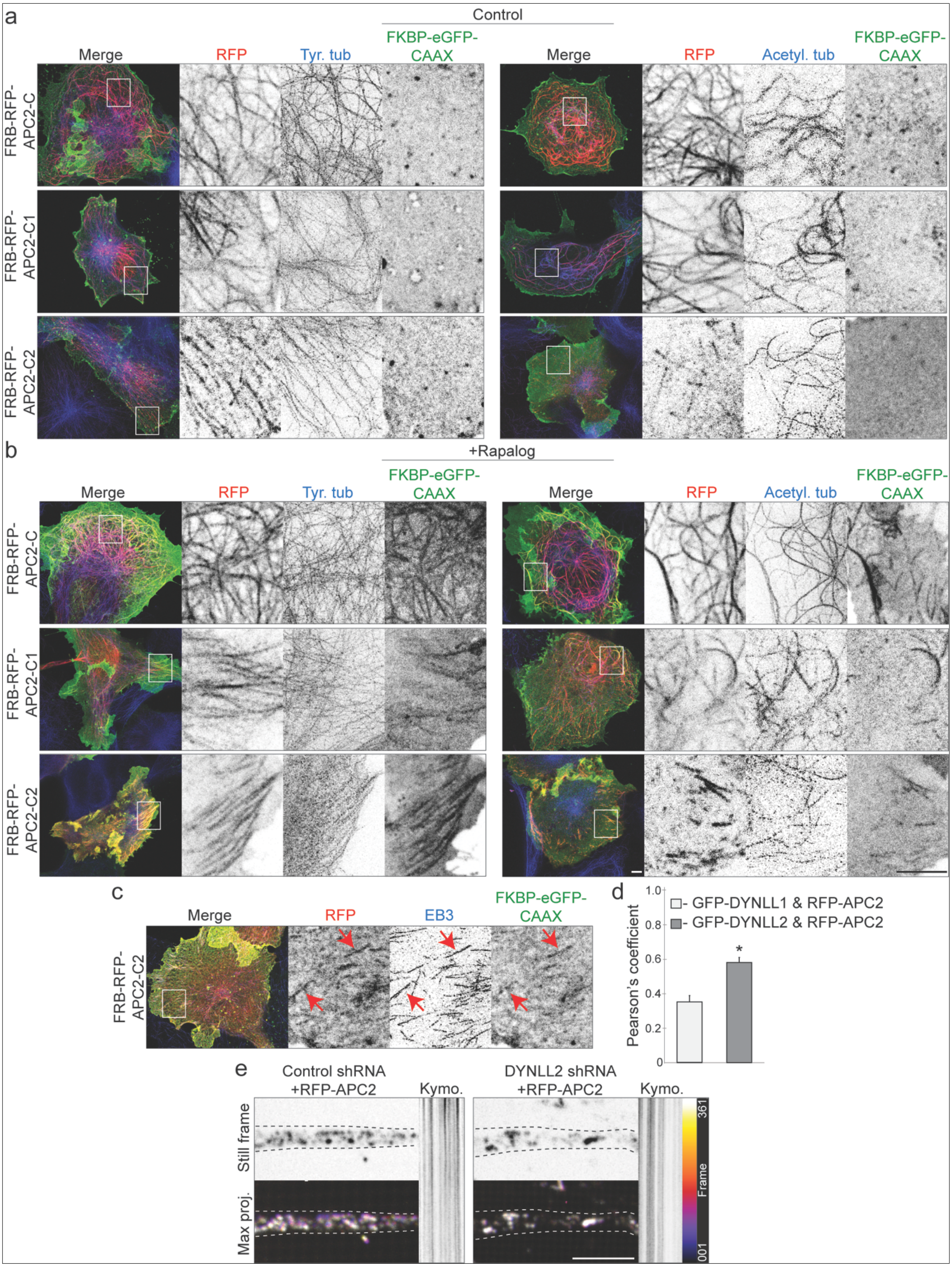

Supplementary Figure 4. Mechanism of C-terminus microtubule binding. **a.** Cos7 cells were transfected with FKBP-eGFP-CAAX and either FRB-RFP-APC2-C, FRB-RFP-APC2-C1 or FRB-RFP-APC2-C2 and fixed 24 h later after 1 h of control treatment. Cells were immunostained for tyrosinated or acetylated tubulin. **b.** Cos7 cells were transfected with FKBP-eGFP-CAAX and either FRB-RFP-APC2-C, FRB-RFP-APC2-C1 or FRB-RFP-APC2-C2 and fixed 24 h later after 1 h of rapalog treatment. Cells were immunostained for tyrosinated or acetylated tubulin. **c.** Cos7 cells were transfected with FKBP-eGFP-

CAAX and FRB-RFP-APC2-C2 and fixed 24 h later after 1 h of rapalog treatment. Cells were immunostained for EB3. **d.** Neurons were transfected with RFP-APC2 and either GFP-DYNLL1 or GFP-DYNLL2 for 24 h and fixed at DIV 15. Pearson's coefficient was quantified from 15 μm sections of proximal dendrite measured from max 2 dendrites per neuron (n=19 areas, N=2, Independent Samples T-test). **e.** Neurons were transfected with either control or DYNLL2 shRNA and RFP-APC2 and imaged 48 h later at DIV 15. Representative still frames, color coded maximum projections and kymographs are shown from 3 min movies taken every 0.5 s. Graphs represent mean ± SEM. * $p<0.05$. Scale bars are 5 μm.
