# Supplementary material for: APC2 controls dendrite development by promoting microtubule dynamics
Source: Nat Commun. 2018 Jul 17;9:2773. doi: 10.1038/s41467-018-05124-5 (PMC6050278; doi:10.1038/s41467-018-05124-5)
Supplement: Supplementary file 3 — Description of Additional Supplementary Files [file 41467_2018_5124_MOESM3_ESM.pdf]

Description of Additional Supplementary Files

File Name: Supplementary Movie 1
Description: Whole microtubule cytoskeleton translocation in dendrites of control and APC2-depleted neurons (related to Fig. 4c). Translocation of photoactivated tubulin region was imaged in dendrites of neurons co-expressing RFP-tubulin with either control or APC2 shRNA. Surface plots correspond to PA-GFP-tubulin channel.

File Name: Supplementary Movie 2
Description: RFP-APC2 C-terminus driven recruitment of microtubules to the cell membrane upon addition of rapalog (related to Fig. 6e). Cos7 cells expressing FKBP-linked membrane-bound domain together with FRB-linked APC2 C-termini truncations were imaged for 40 min after addition of rapalog. Membrane displacement corresponds to microtubule recruitment.
